# Segmented variable-frequency ultrasound synergistic hot-air drying of *Rhubarb*: Effect on drying characteristics and quality and thermal analysis

**DOI:** 10.1016/j.ultsonch.2024.106986

**Published:** 2024-07-09

**Authors:** Xinyu Ying, Fangxin Wan, Tongxun Wang, Zepeng Zang, Yanrui Xu, Bowen Wu, Xiaoping Yang, Xiaopeng Huang

**Affiliations:** College of Mechanical and Electronical Engineering, Gansu Agricultural University, Lanzhou 730070, China

**Keywords:** ANSYS Workbench, Constant-frequency ultrasound, Free anthraquinones, *Rhubarb* slice, Variable-frequency ultrasound

## Abstract

This study employed segmented variable-frequency ultrasound synergistic hot-air drying (SVFU-HAD) for *Rhubarb* slices, selected two sets of time nodes for frequency conversion (60 min, 120 min, and 90 min, 150 min), and two sequences of frequency conversion (high-frequency to low-frequency, and low-frequency to high-frequency). It aimed to investigate the effects of SVFU-HAD on the drying characteristics, quality, and heat transfer of *Rhubarb* slices. The findings indicated that segmented variable-frequency ultrasound has advantages in increasing drying rate and improving uniformity of cavitation effects compared to constant-frequency ultrasound. Analysis of physical properties revealed that the rehydration performance of dried products subjected to ultrasonic variable-frequency treatment (90 min, 150 min) according to the drying rate was better (*RR* > 3.3). The transition mode from high-frequency to low-frequency in variable-frequency ultrasonic treatment contributes to maintaining the overall color of *Rhubarb*. Analysis of chemical properties unveiled that *Rhubarb* treated with 40 kHz (0 min)–28 kHz (60 min)–25 kHz (120 min) segmented variable-frequency ultrasound contained overall higher levels of tannins, dianthrones and free anthraquinones content, which exceeded the average values by 3.24%, 26.65%, and 14.42%, respectively. In addition, thermal analysis results based on ANSYS Workbench software demonstrated that the drying uniformity of SVFU-HAD is superior to that of hot-air drying and constant-frequency ultrasound synergistic hot-air drying (CFU-HAD). Overall, the SVFU-HAD method employed in this study presents an innovative approach to ultrasound synergistic hot-air drying research with promising potential for enhancing the efficiency and quality characteristics of *Rhubarb* slices.

## Highlights

• Technological innovation of segmented variable-frequency ultrasound-assisted drying.
• Simulate the drying heat transfer process using ANSYS software.
• Variable-frequency ultrasound can enhance cavitation and heat transfer uniformity.
• The optimal drying conditions are 40 kHz (0 min)–28 kHz (60 min)–25 kHz (120 min).

## Graphical abstract

**Figure:**
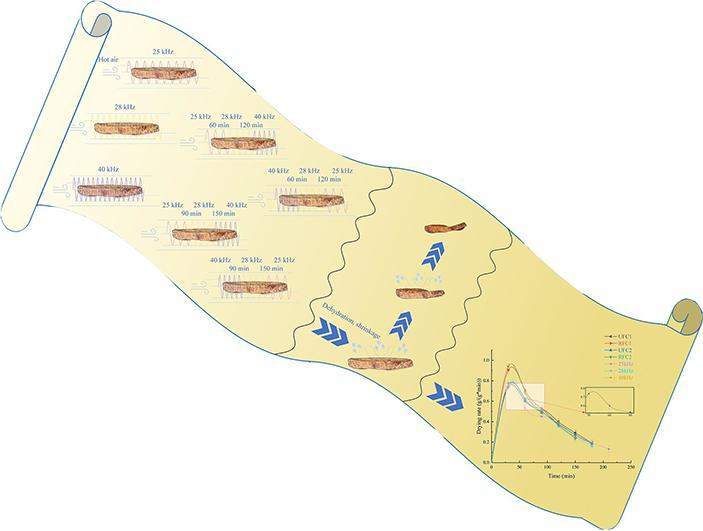

## 1 Introduction

*Rhubarb*, belonging to the genus *Rheum* in the family Polygonaceae, is a collective term encompassing the roots and rhizomes of *Rheum palmatum L.*, *R.tanguticum Maxim.ex Balf.* or *R.officinale Baill.*(medicinal *Rhubarb*) [1]. It is rich in nutrients and contains polyphenols, flavonoids, phytosterols, anthraquinone derivatives, anthraquinone glycosides, tannins, and other substances. Modern pharmacological studies have revealed that *Rhubarb* possesses a significant medicinal value due to its effects, such as inducing laxation, protecting the liver and kidneys, as well as exerting anti-inflammatory and anti-allergic properties [2], [3], [4], [5]. However, due to its high moisture content, improper storage of *Rhubarb* can lead to issues such as rotting, browning, and, importantly, the loss of natural active ingredients such as anthraquinones. The drying process effectively inhibits the proliferation of microorganisms and enzyme activity in *Rhubarb*, thereby reducing the occurrence of physical and chemical reactions during storage. This preservation method significantly enhances the retention of natural active ingredients. Therefore, drying plays a pivotal role in the industrial production of *Rhubarb*.

Currently, the standard drying methods for *Rhubarb* are traditional sun-drying (SD) and smoke drying (SMD). However, SD entails prolonged drying cycles and is susceptible to weather fluctuations, while SMD poses environmental pollution risks and demands substantial labor input [6]. Consequently, there exists a pressing need to investigate a highly efficient and resource-conserving drying method and process for *Rhubarb*. Hot-air drying (HAD) is a viable alternative method, but its development is hindered by issues such as low heat transfer efficiency and extended deceleration period [7]. In agricultural product drying production, the advent of innovative hot air combined drying technology has significantly enhanced overall efficiency and upheld product quality, rendering it a pivotal frontier in drying technology evolution.

Ultrasound technology, renowned for its profound penetration and robust energy transmission capabilities, propagates longitudinal waves in gases, liquids, and solids, as well as transverse waves with shear force within solid mediums [8]. Ultrasound propagation engenders a spectrum of effects spanning thermal, mechanical, chemical, and cavitation phenomena. Cavitation, in particular, catalyzes a range of consequential mechanisms, including radiation force, shock wave propagation, microjet formation, and material deformation [8]. Currently, ultrasound technology is used extensively in food processing, such as cleaning materials, sterilization, and aiding in drying processes. Among these applications, ultrasound-assisted drying stands out as a crucial dewatering method. It accelerates external heat transfer while reducing internal mass transfer resistance [9]. Relevant studies have indicated that ultrasound-assisted drying not only accelerates the drying process but also amplifies the energy production efficiency and biodegradability of wastewater in fruit drying processes [10], [11]. Ultrasound synergistic hot-air drying represents a sophisticated amalgamation wherein ultrasonic waves impart acoustic pressure onto materials, instigating thermal effects and synergistically interacting with hot air currents to induce desiccation [9]. Unlike conventional hot-air drying, this method operates at lower temperatures, rendering it especially conducive to materials harboring thermosensitive natural constituents, thus yielding superior product integrity [9]. Ultrasound synergistic hot-air drying not only accelerates the drying rate and conserves energy but also enhances the quality of dried products. This demonstrates significant potential for widespread application [12]. In the research realm of ultrasound synergistic hot-air drying, studies by Liu et al. [13] have demonstrated that this method can expedite mass transfer within materials while reducing energy consumption. Moreover, Thanompongchart et al. [14] have confirmed that ultrasound synergistic hot-air drying can bolster the moisture evaporation rate of pineapple slices.

In recent years, ultrasound applications in food processing have primarily focused on single-frequency or multiple-frequency constant-frequency ultrasounds. However, a holistic assessment reveals that single-frequency ultrasound drying manifests a sluggish drying rate, feeble ultrasonic cavitation effect, and uneven energy dissipation [15]. In comparison, multi-frequency ultrasound improves the uniformity of the sound field and enhances the cavitation effect over single-frequency ultrasound. Nevertheless, multi-frequency ultrasound consumes more energy, has poor economic efficiency, and causes significant damage to the internal structure of materials [16]. Furthermore, the prolonged emission of constant-frequency ultrasonic waves not only fails to maximize the ultrasonic resonance-induced effects but also needs to improve in enhancing material drying characteristics and quality [17]. Therefore, it is necessary to explore an ultrasound-assisted modality with a more integrated effect. Variable-frequency ultrasound is a mode of ultrasound action in which the frequency is regularly shifted at specific time points. Currently, variable-frequency ultrasound has demonstrated promising applications in optimizing the extraction process of rose polyphenols and facilitating the enzymatic hydrolysis reaction of rapeseed protein [18], [19]. In addition, the utilization of alternating dual-frequency variable-frequency ultrasound in drying pretreatment underscores its high feasibility in the food processing stage [20]. However, there are fewer studies on the synergistic effect of variable-frequency ultrasound with other drying techniques, leaving unexplored whether altering ultrasound frequencies can yield improved drying characteristics and superior quality of dried products. For this reason, this study set the ultrasonic frequency in segments on the basis of the single-frequency test and investigated the effects of segmented variable-frequency ultrasound synergistic hot-air drying (SVFU-HAD) on the drying characteristics of *Rhubarb*, color difference, natural active ingredients, rehydration ratio, microstructure, and the temperature change of the heat transfer process, aiming to delineate a more efficacious ultrasound synergistic hot-air drying process for *Rhubarb* slices.

## 2 Materials and methods

### 2.1 Experimental materials

The experiment selected *Rhubarb* (*Rheum palmatum L.*) grown in Hui County planting base in Longnan City, Gansu Province. Fresh *Rhubarb* of similar size without diseases or pests was chosen. The average initial moisture content of fresh *Rhubarb* was 69.47% ± 0.50% (HKSF-2 rapid moisture analyzer, Wuxi Huake Instrument Co., Ltd., Wuxi, China), and then the *Rhubarb* was stored in a constant temperature and humidity chamber (4 °C) after purchase.

### 2.2 Experimental equipment

**Fig. 1:**
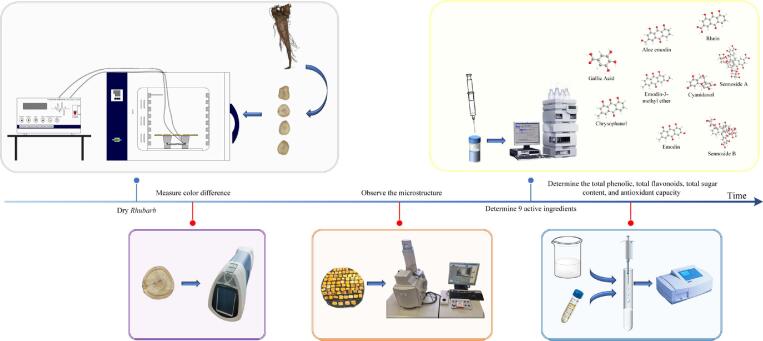
Experimental flowchart.

The experimental flow chart of this study is depicted in Fig. 1. The primary instruments and equipment used are as follows: electrothermal blowing dry box (YQ101-0A-4A, Tianjin Shunnuo Instrument Technology Co., Ltd., Tianjin, China), ultrasonic generator (Shenzhen Dianke Ultrasonic Co., Ltd., Shenzhen, China), ultrasonic transducer (Shenzhen Dianke Ultrasonic Co., Ltd., Shenzhen, China), colorimeter (CR-410, Konica Minolta, Tokyo, Japan), scanning electron microscope (S3400N, Hitachi Scientific Instruments Co., Ltd., Tokyo, Japan), UV–visible spectrophotometer (T6 New Century, Beijing Puxi General Instrument Co., Ltd., Beijing, China), and high-performance liquid chromatograph (Agilent 1100, Agilent Co., Ltd., Palo Alto, USA).

### 2.3 Experimental method

The *Rhubarb* was sliced (horizontally) and measured using a vernier caliper to ensure a thickness range of (4 ± 0.5) mm. Subsequently, the slices were methodically arranged in a random and uniform manner on a drying tray, with each group meticulously weighed to fall within the specified range of (120 ± 1) g (electronic analytical balance, Yuyao Jiming Weighing Verification Equipment Co., Ltd., Yuyao, China). Following a rigorous 20-minute preheating phase, ensuring drying equipment reaches the predetermined parameters, the sliced *Rhubarb* was placed into the ultrasound synergistic hot-air drying equipment. During the drying process, the sample quality is to be measured every 30 min and then promptly returned to the drying chamber to continue drying until the moisture content of the sample reaches the safe storage moisture content of 12.00% [21]. SD group served as the blank control group, with samples placed in an environment characterized by exposure to sunlight and dry ventilation. All experimental samples underwent ultrasound synergistic hot-air drying except for SD. In instances where variable-frequency ultrasound treatment was employed, the ultrasonic frequency underwent precise adjustments at specified time nodes. Each experimental condition was repeated three times, and the results were averaged from three sets of data.

**Fig. 2:**
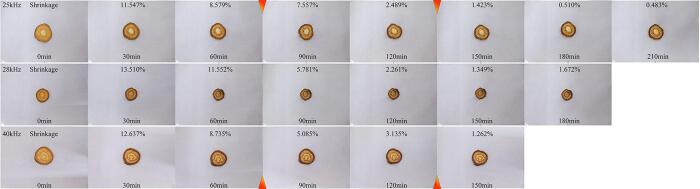
Physical shape changes of the *Rhubarb* drying process under the constant-frequency ultrasound of 25 kHz, 28 kHz, and 40 kHz. (Shrinkage (%) = ((*A*_*0*_−*A*_*1*_)/*A*_*0*_) × 100, *A_0_* is the area of the *Rhubarb* slice in the first 30 min, *A_1_* is the area of the *Rhubarb* slice at the current moment). Symbolize markers of frequency conversion.

**Fig. 3:**
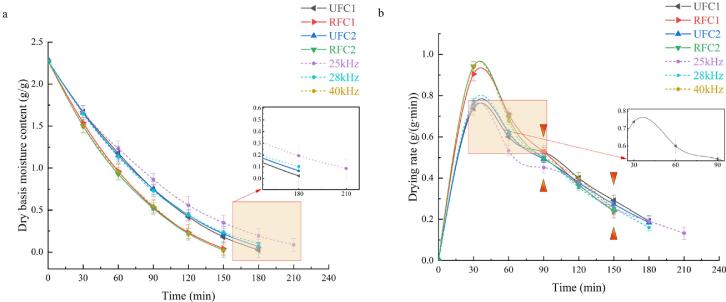
Dry basis moisture content curve (a) and drying rate curve (b) of *Rhubarb* under different ultrasonic frequency conditions.

The preliminary experiments determined that the optimal conditions for ultrasound synergistic hot-air drying of *Rhubarb* slices included a temperature of 55 °C), ultrasonic power of 120 W, and the utilization of both constant-frequency and variable-frequency ultrasound modes. In the constant-frequency mode serving as the conditional control group, frequencies of 25 kHz, 28 kHz, and 40 kHz were tested. The study primarily focuses on investigating the effect of segmented variable-frequency ultrasound on the drying characteristics and quality. The selection of time nodes for frequency conversion is comprehensively based on the consideration of both the shrinkage rate and drying rate during the drying process of *Rhubarb* slices. Fig. 2 illustrates the physical shape changes of *Rhubarb* at different time points during constant-frequency ultrasound treatment, with the corresponding drying rate curve of *Rhubarb* under the same conditions shown in Fig. 3 (b). Based on the comparison of shrinkage rates during the drying process in the control group (Fig. 2), it is evident that the average shrinkage rate of the samples is lowest when subjected to the 25 kHz condition within the initial 0 to 60-minute timeframe of the drying process. Subsequently, during the 60 to 120-minute timeframe, the sample average shrinkage rate is the lowest under 28 kHz conditions. Beyond the 120-minute mark, the sample average shrinkage rate is the minimum under 40 kHz conditions. The contraction reaction induced by drying can affect the moisture stress, leading to cracking and collapse of materials, consequently diminishing the quality of dried products [22], [23]. Therefore, in light of minimizing the impact of shrinkage, a frequency combination with less shrinkage effect is considered, with 60 min and 120 min selected as a pair of time nodes for frequency conversion. From a drying rate perspective, Fig. 3(b) illustrates that during the initial 1/2 stage of the drying process (90 min prior), the drying rate of samples under the 40 kHz condition is notably faster compared to those under 25 kHz and 28 kHz conditions. This highlights a distinct advantage of ultrasonic treatment at 40 kHz. However, after 150 min of drying, the drying rate of samples under 40 kHz ultrasonic action starts to be lower than that under 25 kHz. It can be seen that different ultrasonic frequencies are suitable for *Rhubarb* at different time periods, with 90 min and 150 min being critical nodes where the drying rate undergoes a significant transition. In summary, the time points of 60 min and 120 min were selected as the time nodes for frequency conversion when considering the shrinkage rate, while 90 min and 150 min were selected as the time nodes for frequency conversion when considering the drying rate. These time nodes were utilized to investigate the effects of SVFU-HAD on the drying characteristics and quality of *Rhubarb*. The sequence of frequency conversion comprises two modes: the upscaled-frequency ultrasound (UFC) from low-frequency to high-frequency and the reduced-frequency ultrasound (RFC) from high-frequency to low-frequency. The specific variable-frequency processing schemes are outlined in Table 1.

**Table 1:** Experimental schemes for SVFU-HAD.

	Time nodes of frequency conversion	Variable-frequency schemes	
1	60 min and 120 min	25 kHz (0 min)–28 kHz (60 min)–40 kHz (120 min)	UFC1
2	60 min and 120 min	40 kHz (0 min)–28 kHz (60 min)–25 kHz (120 min)	RFC1
3	90 min and 150 min	25 kHz (0 min)–28 kHz (90 min)–40 kHz (150 min)	UFC2
4	90 min and 150 min	40 kHz (0 min)–28 kHz (90 min)–25 kHz (150 min)	RFC2

### 2.4 Calculation of drying parameters

#### 2.4.1 Dry basis moisture content

The dry basis moisture content in the drying process of *Rhubarb* was calculated according to Eq. (1) [24]:

(1) $$M_{\text{t}}=\left(m_{t}-m_{g}\right)/m_{g}$$

Where *M_t_* is the dry basis moisture content of *Rhubarb* at time *t*, g/g; *m_t_* is the quality of *Rhubarb* at time *t*, g; *m_g_* is the quality of *Rhubarb* dry matter, g.

#### 2.4.2 Drying rate

The drying rate, representing the change in moisture content of the *Rhubarb* sample over each time interval during the drying process, can be calculated as follows Eq. (2) [25]:

(2) $$V_{R}=\left(M_{t_{1}}-M_{t_{2}}\right)/\left(t_{2}-t_{1}\right)$$

Where *V_R_* is the drying rate of the *Rhubarb*, g/(g∙min); *t_1_* and *t_2_* are any drying time, min; $M_{t_{1}}$ is the dry basis moisture content of *Rhubarb* at time *t_1_*, g/g; $M_{t_{2}}$ is the dry basis moisture content of *Rhubarb* at time *t_2_*, g/g.

### 2.5 Ultrasonic cavitation field

The ultrasonic cavitation field distribution was assessed using the dyeing method [26] under various conditions. A 5 mg/mL aqueous solution of methylene blue was prepared and added to the material tray while the coated paper was positioned horizontally. Subsequently, constant-frequency ultrasound at 25 kHz, 28 kHz, and 40 kHz, as well as variable-frequency ultrasound, were applied successively. Each frequency of ultrasound was allowed to act for 1 min before the coated paper was removed for observation.

### 2.6 Rehydration ratio

The dried *Rhubarb* samples were subjected to a controlled temperature water bath at 40 °C) for a duration of 130 min, following which they were removed. The surface moisture of the *Rhubarb* slices was repeatedly absorbed using absorbent paper until the weight remained constant. The weight of the *Rhubarb* was then measured. The formula for calculating the rehydration ratio is as follows Eq. (3) [27]:

(3) $$\mathrm{RR}=\frac{M_{f}}{M_{0}}$$

Where *RR* is the rehydration ratio; *M_f_* is the weight of dried *Rhubarb* products after rehydration, g; *M_0_* is the weight of dried *Rhubarb* products before rehydration, g.

### 2.7 Microstructure

Under the premise of protecting the observation surface, dried samples of *Rhubarb* approximately 6 mm × 4 mm × 3 mm in size were taken and pasted onto a metal sample stage using conductive adhesive. Two 60-second coating treatments were then performed using ion sputtering. The resulting microstructures of *Rhubarb* were observed under various conditions using an S3400N scanning electron microscope at a magnification of 300 times.

### 2.8 Color difference

The CR-410 colorimeter was adopted to detect the color of dried samples under different drying methods. The total color difference Δ*E* was employed to indicate the variation in color between the tested samples and the fresh sample, which was calculated following Eq. (4) [28]:

(4) $$\Delta E=\sqrt{{\left(L^{*}-L_{0}^{*}\right)}^{2}+{\left(a^{*}-a_{0}^{*}\right)}^{2}+{\left(b^{*}-b_{0}^{*}\right)}^{2}}$$

Where L*, a*, and b* represent the lightness value, red-green value, and yellow-blue value of dried *Rhubarb* samples, respectively. L*_0_, a*_0_, and b*_0_ represent the brightness value, red-green value, and yellow-blue value of the *Rhubarb* fresh sample, respectively. Δ*E* represents the total color difference between the dried samples and the fresh sample.

### 2.9 Bioactive compounds

#### 2.9.1 Preparation of sample extracts

The dried *Rhubarb* samples were pulverized and sieved through a 60-mesh screen. Approximately 0.5 g ± 0.05 g of the resulting powder was weighed and placed into a 50 mL conical centrifuge tube, followed by the addition of 25 mL of 75% ethanol (V/V). The tube was then positioned in a light-protected shaker and continuously oscillated for 48 h. Following this period, the mixture was centrifuged for 10 min at a speed of 3000 rpm and a temperature of 26 °C.

#### 2.9.2 Determination of total phenolic content

Following the methodology outlined by Beato et al. [29], adhering to the Folin-Ciocalteu reagent method, the total phenolic content of the *Rhubarb* samples was assessed. Specifically, 10 μL of the extract was aspirated, followed by the sequential addition of 2.0 mL of 10% Folin-Ciocalteu reagent and 1.0 mL of 7.5% Na_2_CO_3_. During the reaction process, the test tube solution changed from the initial extract color to yellow upon the addition of Folin-Ciocalteu. Subsequently, after the addition of Na_2_CO_3_, the reaction mixture was placed in a water bath at 37 °C) in the dark for 1 h to allow for a complete reaction, during which the reaction solution gradually turned blue-gray. In order to establish the standard curve of total phenols, gallic acid was employed as the reference standard:

$$C_{1}=0.0086A-0.000023$$

The formula for calculating the total phenolic content is as follows Eq. (5):

(5) $$\mathrm{TPC}=(C_{1}\times V\times D_{1})/M$$

Where *A* is the absorbance value of the sample solution; *C_1_* is the concentration of total phenols, mg/mL; *TPC* is the total phenolic content, mg/g·DW; *M* is the dry matter weight of the weighed *Rhubarb*, g; *V* is the volume of the extract, mL; *D_1_* is the dilution multiple of the extract for the determination of total phenolic compounds content.

#### 2.9.3 Determination of total flavonoids content

Drawing on the measurement method proposed by Ma et al. [30], the total flavonoids content of the samples was determined through the use of the sodium nitrite-aluminum chloride-sodium hydroxide method. Initially, a volume of 100 μL of the extraction solution was taken to determine the total flavonoids content of the *Rhubarb* samples. Sequentially, 2.0 mL of distilled water, 0.3 mL 5.0% NaNO_2_, 0.3 mL 10% AlCl_3_, and 2.0 mL 1 mol/L NaOH were added. During the reaction process, the test tube solution was initially colorless, turned yellow upon the addition of AlCl_3_, and gradually transformed into red upon the addition of NaOH. In order to establish the standard curve for total flavonoids, Catechin was utilized as the reference standard:

$$C_{2}=0.0319A+0.000818$$

The formula for calculating the total flavonoids content is as follows Eq. (6):

(6) $$\mathrm{TFC}=(C_{2}\times V\times D_{2})/M$$

Where *C_2_* is the concentration of total flavonoids, mg/mL; *TFC* is the total flavonoids content, mg/g·DW; *D_2_* is the dilution multiple of the extract for the determination of total flavonoids compounds content.

#### 2.9.4 Determination of total sugar content

The total sugar content of the sample was determined using the phenol–sulfuric acid method, referencing the measurement technique outlined by Dubois et al. [31]. The sample extraction solution of 5 μL was placed in a test tube, followed by the sequential addition of 1.0 mL 9.0% phenol solution and 3.0 mL concentrated sulfuric acid. After thorough mixing and reaction, the test tube solution changed from colorless to reddish-orange. To establish a reliable standard curve for quantifying the total sugar content, sucrose was utilized as the reference standard:

$$C_{3}=0.0179A-0.000022$$

The formula for calculating the total sugar content is as follows Eq. (7):

(7) $$\mathrm{TSC}=(C_{3}\times V\times D_{3})/M$$

Where *C_3_* is the concentration of total sugar, mg/mL; *TSC* is the total sugar content, mg/g·DW; *D_3_* is the dilution multiple of the extract for the determination of total sugar content.

#### 2.9.5 Determination of antioxidant capacity

The antioxidative capacity of the sample was determined using the DPPH method, employing the protocol outlined by Nencini et al. [32]. The inhibitory rate of *Rhubarb* samples was determined by adding 5 μL of the extraction, followed by the addition of 3.0 mL of 10^−4^ mol/L DPPH. The reaction should then be allowed to oscillate in darkness at room temperature for 30 min. During the reaction, the solution in the test tube gradually changed from purple to a lighter shade. A lighter color suggests a higher inhibition rate, signifying a stronger antioxidative capacity in the sample.

The Inhibitory rate was calculated by Eq. (8):

(8) $$I\left(\%\right)=\left[\left(A_{0}-A\right)/A_{0}\right]\times 100$$

Where *I* represents the inhibitory rate of the sample solution; *A_0_* represents the absorbance value of the solution without the sample.

### 2.10 Natural active ingredients

Preparation of HPLC sample extract [33]: accurately weigh 0.5 ± 0.005 g of *Rhubarb* powder passed through a 60-mesh sieve. Place it in a conical centrifuge tube and add precisely 25 mL of 60% methanol (V/V). Perform ultrasonic extraction for 1 h (at a frequency of 40 kHz and power of 210 W), followed by centrifugation for 10 min (at a temperature of 26 °C and a speed of 3000 rpm). Filter the extract (using a 0.45 μm microporous membrane) and transfer 0.5–1.5 mL into a 2 mL vial for the sample bottle.

Preparation of single reference standards: the preparation of nine single reference standards was conducted to determine the retention times of nine natural active ingredients. Single reference standard stock solutions were prepared for gallic acid, cyanidanol, sennoside A, sennoside B, aloe emodin, rhein, emodin, chrysophanol, and emodin-3-methyl ether, each attaining a purity of ≥98% and a concentration of 1 mg/mL. 3 mg of each compound were dissolved in 3 mL of HPLC methanol. Subsequently, 0.3 mL of each single reference standard stock solution was combined with 0.7 mL of HPLC-grade methanol to yield nine single reference standard solutions, each boasting a concentration of 0.3 mg/mL.

Preparation of mixed reference standards: nine concentrations of mixed reference standards were prepared to establish a standard curve by plotting the relationship between the different concentrations and the corresponding peak areas measured by HPLC. Firstly, 1 mL aliquots of each of the nine single reference standard stock solutions were amalgamated in a test tube and shaken to generate a composite reference standard solution 1, with a concentration of 0.111 mg/mL for each constituent ingredient. Subsequently, composite reference standard solution 2 was formulated by combining 1 mL of composite reference standard solution 1 with 1 mL of HPLC-grade methanol, yielding a concentration of 0.056 mg/mL for each functional ingredient. This process was iterated to generate composite reference standard solutions 3, 4, 5, and 6, ensuring a comprehensive range of concentrations for subsequent analysis.

Aglient1100 HPLC chromatography conditions [33]: chromatographic column: Inertsil ODS C_18_ (250 mm × 4.6 mm, 5 μm). Mobile phase: acetonitrile (B)-0.05% phosphoric acid solution (V/V) (D). Gradient elution procedures: 0–5 min, 95%–89% D, 5–10 min, 89%–85% D, 10–14 min, 85%–75% D, 14–20 min, 75%–35% D, 20–25 min, 35%–30% D, 25–30 min, 30%–25% D, 30–34 min, 25%–20% D, 34–36 min, 20%–95% D, 36–38 min, 95%–95% D. Detecting wavelength: 280 nm.

### 2.11 Thermal simulation of drying process

As the drying process progresses, internal temperature variations occur within the *Rhubarb* slices. This study aimed to examine the influence of ultrasound on heat transfer in materials and to visually depict the uniformity of hot-air drying and ultrasound synergistic hot-air drying. A heat transfer model was developed using the mechanical module of ANSYS Workbench software to achieve this. The model was designed to simulate the temperature dynamics of *Rhubarb* slices under different drying conditions and time intervals, thereby illustrating the temperature changes during the drying process.

The modeling process was based on the following assumptions: (1) The wall of the electrothermal blowing dry box serves as an insulator with no heat dissipation. (2) *Rhubarb* is treated as an isotropic material. (3) Apart from the heat required for water evaporation from *Rhubarb* slices, all other heat is used for material heating. (4) Neglecting the impact of power loss heat from the ultrasonic transducer on the slicing of *Rhubarb*. The thermal supply and dissipation within individual material systems adhere to the law of energy conservation, which can be described by Eq. (9):

(9) $$\rho C_{p}\frac{\partial T}{\partial t}=Q_{1}-Q_{2}+Q_{3}$$

The specific modeling process is outlined as follows.

Firstly, the 3D model of *Rhubarb* was established, material properties were defined, automatic mesh generation was performed, and the initial material temperature was set.

Next, boundary conditions for hot air convection were established. Newton's law of cooling was applied to describe the convective heat transfer in hot-air drying. According to this law, in forced convection scenarios, the rate of heating or cooling of an object is primarily influenced by the ambient temperature rather than its specific physical and chemical properties. The fundamental formula is represented by Eq. (10) [34]:

(10) $$Q_{1}=\overset{¯}{h}\left(T_{1}-T_{0}\right)$$

The drying process involves mass transfer, where the heat dissipation required for moisture evaporation within *Rhubarb* slices, denoted as Q_2_, must be considered in simulations to emulate actual conditions. Each drying process is subject to the superimposition of the Heat Flux environment. The formula for calculating Q_2_ is provided as Eq. (11) [35]:

(11) $$Q_{2}=\frac{\partial w}{s\partial \tau }\left(1.88T_{1}+2492\right)-4.187T_{0}\frac{\partial w}{s\partial \tau }$$

Where *w* is the accumulated water evaporation during the drying period, 2492 kJ/kg is the approximate value of water vaporization heat. 4.187*T_0_w* is the heat brought into the system by moisture in the material, the value of which is relatively small and can be neglected. Eq. (10) is simplified to Eq. (12):

(12) $$Q_{2}=\frac{\partial w}{s\partial \tau }\left(1.88T_{1}+2492\right)$$

Furthermore, a model of ultrasound-assisted drying can be established on the basis of the hot-air drying environment. According to the thermal mechanism of ultrasound, the waves absorbed by the material during the ultrasonic propagation process convert the acoustic energy of ultrasound into internal heat energy Q_3_ through mechanical action. The calculation formula is as shown in Eqs. (13)–(15) [8]:

(13) $$Q_{3}=2\alpha I$$

The calculation formula for acoustic energy density *I* is Eq. (14):

(14) $$I=\frac{P_{A}^{2}}{2\rho c}$$

The calculation formula for acoustic pressure amplitude *P_A_* is Eq. (15):

(15) $$P_{A}=2\pi f\rho cA$$

It is worth noting that the variable-frequency ultrasound in this section needs to consider different ultrasonic frequencies at different stages. The meanings and units of the variables involved in Eqs. (9)–(15) are shown in Table 2.

**Table 2:** List of symbols.

Symbols	Meanings	Units
*C_p_*	Specific heat capacity	J /kg/°C
*Q_1_*	Heat flux density	W/m^2^
*h*	Convective heat transfer coefficient	W/m^2^/°C
*T_1_*	Environmental temperature	°C
*T_0_*	*Rhubarb* surface temperature	°C
*Q_2_*	Heat required to evaporate water from *Rhubarb* slices	W/m^2^
*I*	Acoustic energy density	W/m^2^
*t*	*Rhubarb* drying time	s
*s*	Single piece area of *Rhubarb*	m^2^
*α*	Ultrasound absorption coefficient in *Rhubarb*	
*f*	Ultrasonic frequency	Hz
*ρ*	*Rhubarb* density	kg/m^3^
*c*	Ultrasound propagation velocity in *Rhubarb*	m/s
*P_A_*	Acoustic pressure amplitude	N/m^2^
*A*	Ultrasonic amplitude (transducer and material tray assembly)	m
*Q_3_*	Heat absorbed from ultrasonic conversion per unit volume of *Rhubarb* per second	W/m^3^
*w*	Evaporation of water	kg

### 2.12 Statistical processing and analysis

The experimental data refinement was carried out using Microsoft Excel (Redmond, WA, USA), OriginLab (Northampton, MA, USA), and SPSS (Chicago, IL, USA). The data results were analyzed using one-way analysis of variance (ANOVA), subjected to the Duncan test and homogeneity of variance test, with the mean ± standard deviation represented. When *P* < 0.05, differences in the mean values of the data were considered statistically significant.

## 3 Results and discussion

### 3.1 Effect of SVFU-HAD on the drying characteristics of *Rhubarb* slices

The drying characteristic curves of *Rhubarb* slices under the conditions of three constant-frequency ultrasound synergistic hot-air drying (CFU-HAD) and four SVFU-HAD are shown in Fig. 3. As illustrated, within constant-frequency ultrasound drying, the highest drying rate occurs at 40 kHz, whereas the lowest is observed at 25 kHz. Furthermore, four types of segmented variable-frequency ultrasound drying exhibit reduced drying times compared to 25 kHz constant-frequency ultrasound drying. At 180 min, the dry basis moisture content of samples treated with two types of UFC was lower than that of samples treated with 25 kHz and 28 kHz constant-frequency ultrasonic treatment (Fig. 3(a)), indicating that samples treated with UFC reached the safe dry basis moisture content faster than those treated with 25 kHz and 28 kHz constant-frequency ultrasonic treatment. This could be attributed to the fact that variable-frequency ultrasound is more effective than constant-frequency ultrasound in promoting the formation of micropores and increasing the rate of dehydration [20]. The drying time of samples subjected to the action of two different RFC and 40 kHz constant-frequency ultrasound was found to be essentially equivalent, with no significant difference in the average drying rate among the three treatments (*P* > 0.05). The slope of the drying rate during the deceleration period serves as a gauge for the rate of decline in the drying rate, with a larger slope indicating a swifter decrease and a smaller slope suggesting a more gradual decline. Under the UFC1 drying conditions, the drying rate slope between 30 min and 60 min is 1.878 times that between 60 min and 90 min. Under RFC2 drying conditions, the drying rate slope between 60 min and 90 min is 1.485 times that between 90 min and 120 min. This indicates that variable-frequency ultrasound, to some extent, attenuates the decreasing trend of the drying rate. Xu et al. [18] have also found that variable-frequency ultrasound can more effectively promote mass transfer, primarily due to the replacement of the pulse intervals of constant-frequency ultrasound by another ultrasound frequency.

**Fig. 4:**
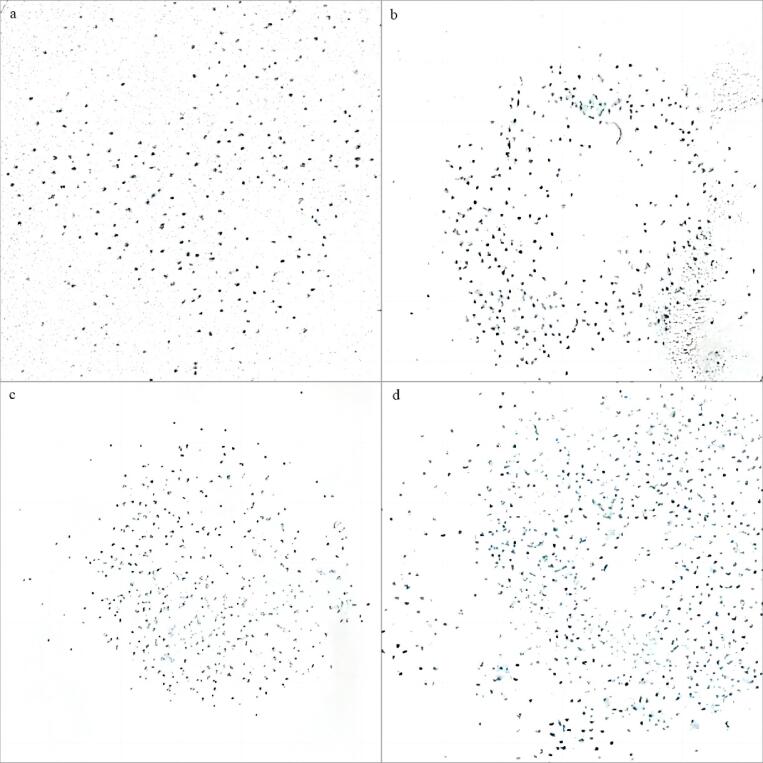
Dye adhesion situation on coated paper under different ultrasonic frequency conditions.

This study utilized the dyeing method to characterize the cavitation field in order to investigate the distinct effects of constant-frequency and variable-frequency ultrasonic cavitation. As depicted in Fig. 4, an increase in ultrasonic frequency results in finer and denser cavitation effect points. Moreover, compared to constant-frequency ultrasound, variable-frequency ultrasound yields a more uniform cavitation field with a higher density of cavitation effect points. This finding aligns with previous studies utilizing the aluminum foil corrosion method and hydrophone measurements [18], [36]. Under variable-frequency ultrasound conditions, bubbles of different frequencies within the slices of *Rhubarb* occur, creating a vibrational phenomenon, activating more bubble activity and forming a larger effective cavitation area. Therefore, the cavitation effect generated by variable-frequency ultrasound is superior to constant-frequency ultrasound, optimizing the ultrasonic excitation effect, thus further affecting product quality.

### 3.2 Effect of different drying conditions on drying quality

#### 3.2.1 Effect of different drying conditions on rehydration ratio

**Fig. 5:**
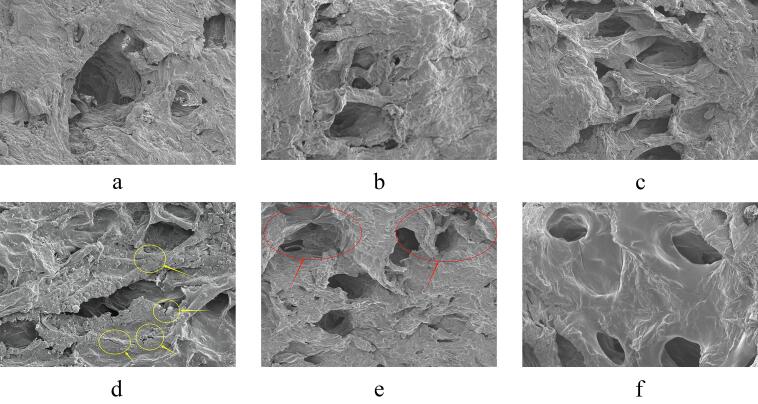
Microstructure images under different processing conditions of *Rhubarb* at 25 kHz (a), 28 kHz (b), 40 kHz (c), sun-drying (d), RFC1 (e), and RFC2 (f) (Yellow markers indicate cracked and damaged microstructures, red markers indicate shrunk and deformed microstructures).

Many scholars believe that the rehydration ratio of dried products is primarily related to the varying degrees of damage or changes in microstructure due to different stresses within the internal cells and structures during the drying process [37]. Table 3 provides insights into the rehydration ratio of *Rhubarb* samples under different drying conditions, while Fig. 5 illustrates the microstructure of *Rhubarb* samples under different drying conditions. Observing Table 3 reveals that the rehydration ratio of *Rhubarb* slices increases with the rise of ultrasonic frequency. This phenomenon is likely attributed to the enhanced mechanical action on the *Rhubarb* slices with increasing ultrasonic frequency, leading to continuous expansion and contraction of cell tissues, thereby promoting the formation of micro-pores [38]. Consequently, the cell tissues of *Rhubarb* slices become loose (as shown in Fig. 5(a)–(c)), which facilitates the absorption of more water, thus improving the rehydration ratio. Also, from Table 3, it is evident that the samples subjected to RFC2 variable-frequency processing exhibit the best rehydration ratio. From a microscopic perspective, as analyzed in Fig. 5(f), under this ultrasonic processing condition, the damage to the sample's microporous structure is relatively minor. Research by Azam et al. [39] also indicates that samples subjected to triple-frequency sequential processing experience less mechanical damage. In comparison, samples processed with RFC1 and SD exhibited poorer rehydration ratios. Fig. 5(d) and (e) depict distinct cracks in the SD samples, while noticeable shrinkage deformation is observed in samples processed with RFC1, likely contributing to their poor rehydration ratios. Furthermore, in samples subjected to variable-frequency ultrasonic treatment, the two groups of samples with 90 min and 150 min as the time nodes for frequency conversion demonstrated enhanced rehydration properties. This may be due to the fact that the variable-frequency ultrasonic combination according to the drying rate dividing frequency helped to improve the effect of ultrasound on the structure of *Rhubarb* slices, which resulted in the improvement of the rehydration ratio of the dried *Rhubarb* products.

**Table 3:** Rehydration ratios of *Rhubarb* samples under different drying conditions.

Drying conditions	Rehydration ratios
Sun-drying	2.852 ± 0.223^e^
UFC1	3.255 ± 0.100^ab^
RFC1	2.897 ± 0.024^de^
UFC2	3.328 ± 0.061^ab^
RFC2	3.377 ± 0.051^a^
25 kHz	3.044 ± 0.069^cd^
28 kHz	3.143 ± 0.082^bc^
40 kHz	3.343 ± 0.090^a^

#### 3.2.2 Effect of different drying conditions on color appearance

The parameter *ΔE* serves as an objective indicator of the appearance quality of dried products. A lower *ΔE* value corresponds to a higher appearance quality of dried products, thereby increasing consumer purchasing desire. Furthermore, the traits of medicinal herbs serve as an objective reflection of intrinsic quality. Consequently, the *ΔE* value, to a certain degree, elucidates the relative abundance of natural bioactive constituents nestled within *Rhubarb*.

**Fig. 6:**
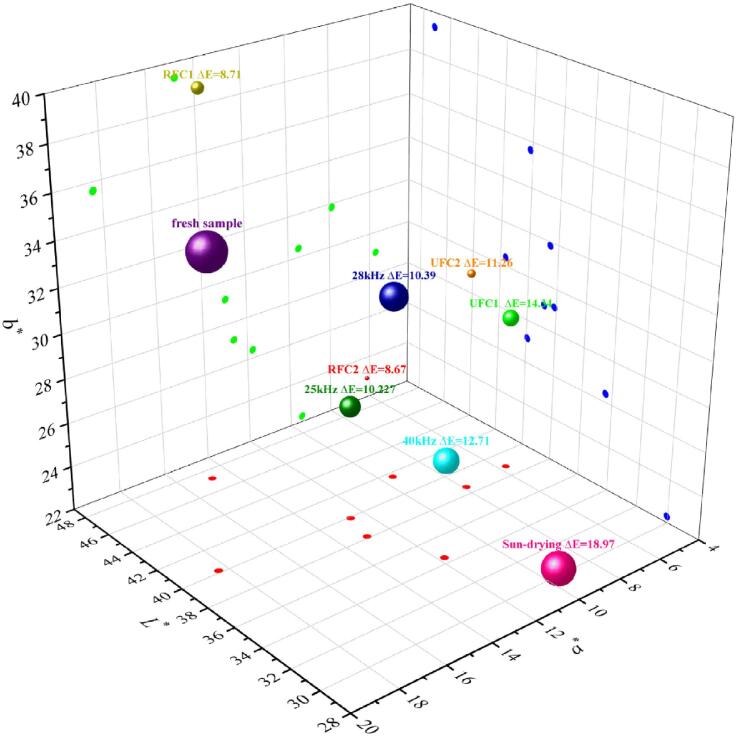
3D scatter plot of *Rhubarb* color difference for different ultrasonic frequency conditions.

As illustrated in Fig. 6, the distance value between the points of dried *Rhubarb* products under different conditions and fresh sample point in the space Cartesian coordinate system corresponds to the *ΔE* value of the dried products. Notably, the *ΔE* value demonstrates a general uptrend with the escalation of ultrasonic frequency under constant-frequency ultrasound, as can be seen in Fig. 6. This phenomenon can be attributed to the higher ultrasonic frequency, which amplifies the mechanical effects within the *Rhubarb*, resulting in the formation of additional micro-pores [38]. This facilitates the migration of substances within the *Rhubarb*, resulting in the loss of internal pigment components in *Rhubarb*, consequently causing an increase in the *ΔE* value. Furthermore, the *Rhubarb* dried product exhibits negative values for overall Δa* and Δb*, which may be attributed to the fact that anthocyanins and cell sap were released into the intercellular space during the drying process, where anthocyanins turn bluish-green when they encounter the alkaline *Rhubarb* cell sap [40]. The *ΔE* value of dried samples treated by RFC is smaller than that of the dried samples treated by UFC, while the *ΔE* value of SD samples is the largest, which is related to the drying duration. Kowalski et al. [41] also observed that a shorter drying time has a protective effect on the color of dried products. Additionally, it can be observed that the *ΔE* values of dried samples treated with variable frequency at the critical time nodes of drying rate transition (90 min and 150 min) are smaller compared to those treated with variable frequency at the critical time nodes of shrinkage rate transition (60 min and 120 min). It is noteworthy that the dried samples treated with RFC1 show the smallest values for Δa* (−3.56) and Δb* (3.74), while those treated with RFC2 exhibit the smallest value for ΔL* (−2.03). This indicates that the variable-frequency method of RFC can better preserve the color of *Rhubarb*.

#### 3.2.3 Effect of different drying conditions on TPC, TFC, TSC, and antioxidant capacity

**Fig. 7:**
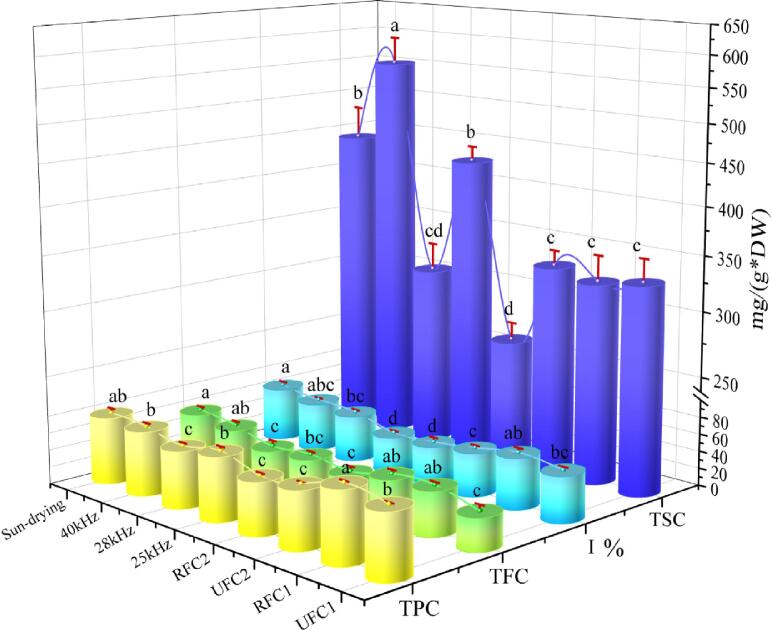
Total phenolic, total flavonoid, total sugar content, and antioxidant capacity of *Rhubarb* under different ultrasonic frequency conditions.

The total phenolic content, total flavonoids content, total sugar content, and antioxidant capacity play a significant role in the efficacy of *Rhubarb*. Fig. 7 delineates these parameters across varied ultrasonic frequency conditions. Remarkably, under RFC1 conditions, the total flavonoids content and antioxidant capacity of *Rhubarb* samples are only slightly lower than those of SD samples, with total phenolic content reaching as high as 82.215 mg/g, exceeding that of SD samples. It is evident that the RFC1 variable-frequency condition has a positive effect on ameliorating the total phenolic content, total flavonoids content, and antioxidant capacity of *Rhubarb* through ultrasound synergistic hot-air drying. This phenomenon can be attributed to the higher ultrasonic frequency in the initial stage, which increases microporosity, thereby facilitating the subsequent release of total phenols and total flavonoids. Subsequently, in the second and third stages, the prolonged application of low-frequency ultrasound enables deeper tissue penetration, progressively releasing additional phenolic compounds. In contrast, samples treated with RFC2 and 28 kHz constant-frequency ultrasound exhibited lower total phenolic and total sugar content (60.406 mg/g, 65.928 mg/g, and 260.327 mg/g, 305.322 mg/g, respectively), samples treated with UFC1 and RFC2 showed lower total flavonoids content (37.139 mg/g, 37.014 mg/g), and samples treated with RFC2 and 25 kHz constant-frequency ultrasound displayed lower antioxidant capacity (41.25%, 43.72%). These findings indicate a significant negative impact of RFC2, UFC1, 28 kHz constant-frequency, and 25 kHz constant-frequency ultrasound treatments on the retention of total phenols, total flavonoids, total sugars, and DPPH free radicals in *Rhubarb*. Under the conditions of RFC2, the total phenolic and total sugar content in dried products are relatively low. This could be due to insufficient low-frequency action time in the later stage, resulting in a decrease in the release rate of total phenolic and total sugar. Nevertheless, Tao et al. [42] found that excessively long low-frequency ultrasonic action time may lead to ingredient degradation. Therefore, it is crucial to balance the action time of different frequencies during the process of frequency conversion. In addition, it was observed that the overall trends in changes of total flavonoids and total phenolic content were generally consistent with the antioxidant capacity, indicating a correlation between total phenolic and total flavonoids content with antioxidant capacity, in line with the research results of Claudia and Zhang et al. [43], [44].

#### 3.2.4 Effect of different drying conditions on the natural active ingredients

**Fig. 8:**
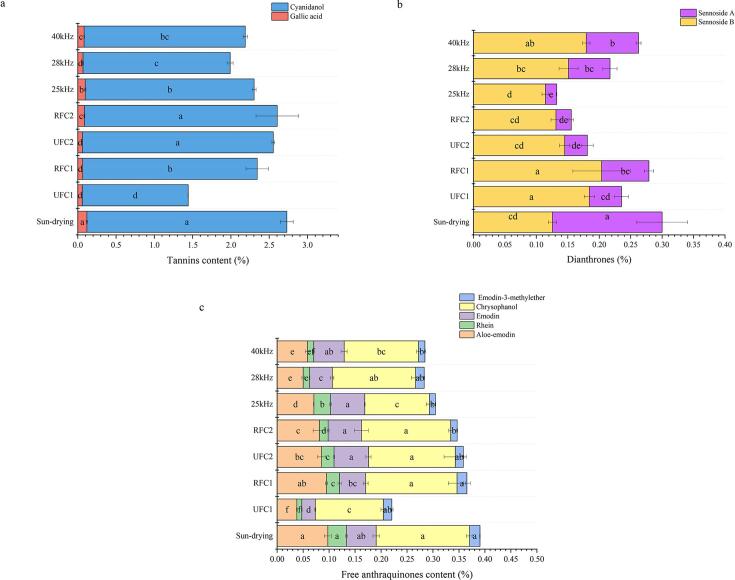
Content of *Rhubarb* tannins (a), dianthrones (b), and free anthraquinones (c) under different ultrasonic frequency conditions.

The natural active ingredients such as free anthraquinones, tannins, and dianthrones in *Rhubarb* exhibit significant biological activities. Among these, free anthraquinones demonstrate antibacterial and anti-inflammatory effects, tannins possess anticoagulant properties, and dianthrones are the primary components responsible for the laxative effects of *Rhubarb* [45], [46], [47], [48]. These three types of components show significant biological activity within *Rhubarb*. Under different ultrasonic frequency conditions, the content of tannins, dianthrones, and free anthraquinones is illustrated in Fig. 8. Upon observation, it is noted that under the conditions of UFC1 treatment, the content of tannins, dianthrones, and free anthraquinones are the lowest, rendering it unsuitable for the drying of *Rhubarb*. Under variable-frequency processing conditions, the average content of tannins, dianthrones, and free anthraquinones are 3.51%, 4.32%, and 10.97% higher, respectively, compared to constant-frequency conditions. This phenomenon is attributed to the heightened cavitation nuclei generation facilitated by variable-frequency ultrasound (seen in Fig. 4), thereby enhancing the dissociation of gallic acid, cyanidanol, and free anthraquinones from their respective complexes. When comparing SVFU-HAD and CFU-HAD with SD experiments, it was observed that the samples with the least loss in tannins, dianthrones, and free anthraquinones were consistently the samples treated with RFC. Especially noteworthy is the minimal loss of dianthrones and free anthraquinones components, processed through RFC1, amounting to 6.95% and 6.45%, respectively. Under this variable-frequency condition, the content of tannins, dianthrones, and free anthraquinones exceed the average values of the other experimental groups by 3.24%, 26.65%, and 14.42%, respectively. It can be observed that the RFC1 treatment mode plays a positive role in retaining the natural active ingredients of *Rhubarb*. This phenomenon may be attributed to the enhanced mutual conversion of the compounds with these natural active ingredients in the RFC1 variable-frequency environment, thereby leading to a higher in their content [49]. Collectively, it can be observed that different drying processes significantly alter the natural active ingredients content of *Rhubarb* slices, with samples treated with RFC1 showing the best overall effect.

### 3.3 Thermal simulation results analysis

**Fig. 9:**
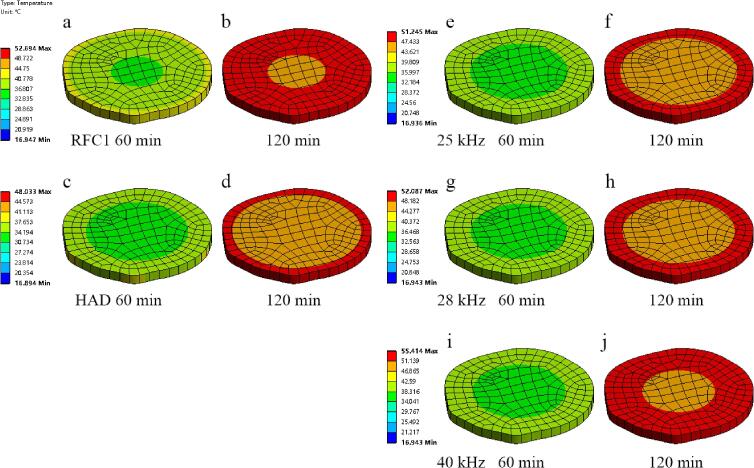
Simulation temperature distribution contours of *Rhubarb* slices under different drying conditions.

**Fig. 10:**
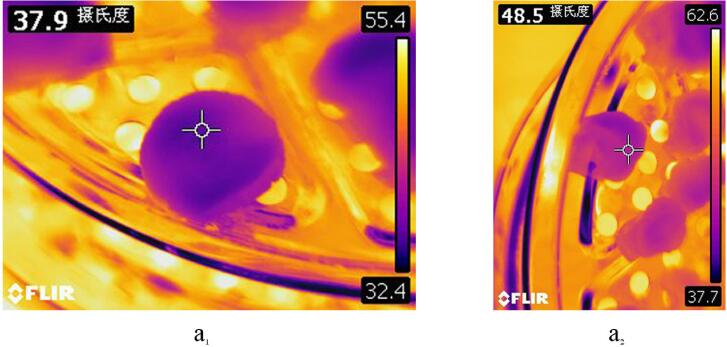
Thermal imaging of *Rhubarb* under RFC1 condition for 60 min (a_1_) and 120 min (a_2_).

**Fig. 11:**
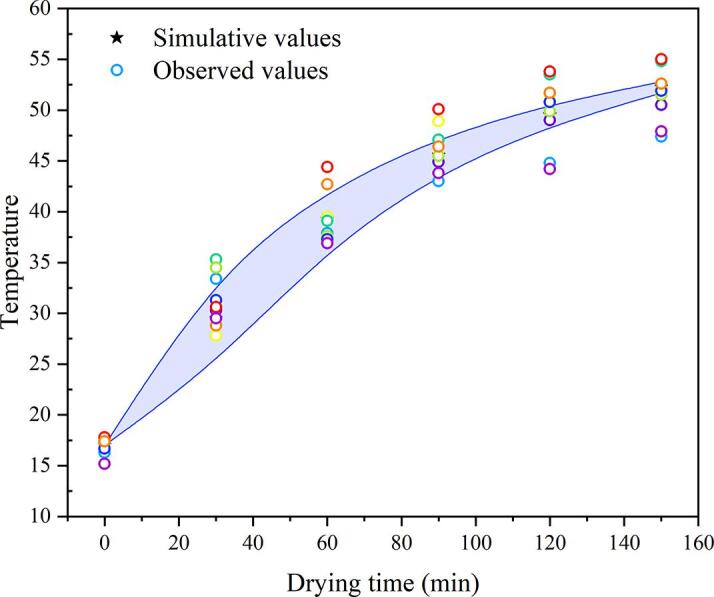
Thermal imaging temperatures and simulated temperatures at different moments of *Rhubarb* under RFC1 condition.

The simulated temperature distribution cloud diagram of the *Rhubarb* drying process under different conditions is shown in Fig. 9. Comparing the simulation results of RFC1 in Fig. 9(a) and (b) with the thermal images of RFC1 obtained through thermal imager (as shown in Fig. 10), it is evident that at 60 min and 120 min, the observed temperatures are 37.9 °C and 48.5 °C, respectively, consistent with the simulated temperature range of *Rhubarb* slices (36.066 °C–41.798 °C and 48.395 °C–50.462 °C. Further analysis of the thermal imaging temperature and simulation temperature at different times of RFC1, as shown in Fig. 11, reveals that the relative error between the average values of simulated and observed temperatures at each time point is within 5%, with a good degree of agreement and the changing trend being basically consistent, indicating the simulation results are reliable under certain conditions. Therefore, it can be seen that the simulation model can be used to simulate and predict the heat transfer phenomenon of ultrasound synergistic hot-air drying under certain conditions, aiding in resolving heat-related issues during the drying process and improving the quality of dried products.

The heat source for ultrasound synergistic hot-air drying of *Rhubarb* involves two main aspects: the convective heat transfer generated by hot air and the ultrasonic thermal effect. The latter entails the propagation of ultrasonic waves within the material, inducing friction within its structure, thus converting acoustic energy into heat energy and consequently elevating the temperature of *Rhubarb*. In contrast to conventional hot-air drying methods, the SVFU-HAD demonstrates an average temperature escalation of 4.436 °C and 5.608 °C at 60 min and 120 min, respectively, as illustrated in Fig. 9. This observed phenomenon can be ascribed to the efficient absorption of ultrasonic energy by *Rhubarb* slices, which accelerates the drying process compared to conventional methodologies. From the maximum and minimum temperature variations in the heat transfer simulation model of the RFC1 synergistic hot-air drying *Rhubarb* shown in Fig. 11, it can be seen that the temperature difference of the drying process gradually increases and then decreases. Furthermore, as observed in Fig. 9, compared to hot-air drying and CFU-HAD, when *Rhubarb* slices are subjected to SVFU-HAD, the overall temperature can reach a relatively stable state more quickly, thereby improving the uniformity of drying heat transfer. This is because, for one thing, the heat of hot-air drying is transferred from the surface of the material to the interior, while the heat in ultrasound-assisted drying acts on the interior of the material. The synergistic effect of these two methods helps in redistributing the temperature, thus improving the uniformity of drying [50], [51]. Additionally, compared to constant frequency ultrasound, variable frequency ultrasound requires less time to reach the voltage amplitude, which can more quickly generate cavitation effects, reduce the moisture diffusion resistance in the material, accelerate the drying efficiency, and improve the uniformity of drying [18].

## 4 Conclusion

This study combined segmented variable-frequency ultrasound with hot-air drying to compare the drying characteristics and quality of *Rhubarb* slices under different constant-frequency and variable-frequency ultrasound treatment conditions. The results indicate that compared to CFU-HAD, SVFU-HAD contributes to optimizing the cavitation effect and enhancing the drying characteristics. In terms of rehydration ratio, the *Rhubarb* dried products treated with 90 min and 150 min as time nodes of frequency conversion demonstrate advantages, with values of 3.328 ± 0.061 and 3.377 ± 0.051, respectively. The utilization of RFC variable-frequency ultrasound treatment can decrease *ΔE* value by at least 14.83%, which contributes to maintaining the color of *Rhubarb*. Among the seven ultrasound synergistic hot-air drying methods, RFC1 demonstrates a more pronounced overall performance in preserving the content of bioactive components, such as TPC and free anthraquinones. Furthermore, this study utilized ANSYS Workbench software to simulate the heat transfer process, demonstrating a good agreement between simulated and observed values with a relative error in average temperature of less than 5%. The simulation results indicate that compared to HAD and CFU-HAD, SVFU-HAD exhibits better drying uniformity. Overall, the RFC1 drying method is more effective in enhancing the drying efficiency and improving the quality of dried products. Combining the aforementioned results indicates that the rational application of segmented variable-frequency ultrasound technology can more effectively harness the ultrasonic effects. This study serves as a compelling testament to the substantial promise of segmented variable-frequency ultrasound in elevating the efficacy of ultrasound synergistic hot-air drying, thereby offering invaluable insights for augmenting the industrial value of ultrasound-assisted drying techniques and optimizing the caliber of dried products.

## Funding

This work was supported by the Young Mentor Fund project of Gansu Agricultural University [grant number 0522014] and the Gansu Provincial Science and Technology Plan [grant number 23CXNA0017].

## Declaration of competing interest

The authors declare that they have no known competing financial interests or personal relationships that could have appeared to influence the work reported in this paper.

## Data Availability

The authors confirm that the data supporting the findings of this study are available within the article.
